# Molecular characterization and antiviral activity test of common drugs against echovirus 18 isolated in Korea

**DOI:** 10.1186/1743-422X-8-516

**Published:** 2011-11-11

**Authors:** KwiSung Park, SangGu Yeo, KyoungAh Baek, DooSung Cheon, YoungJin Choi, JoonSoo Park, SooJin Lee

**Affiliations:** 1 Department of Microbiology, Chungcheongnam-Do Institute of Health and Environmental Research, Daejeon, Korea; 2 Division of Enteric and Hepatitis Viruses, Center for Infectious Diseases, National Institute of Health, Korea Center for Disease Control and Prevention, Osong, Korea; 3 Department of Laboratory Medicine, College of Medicine, Soonchunhyang University, Cheonan, Korea; 4 Department of Pediatrics, College of Medicine, Soonchunhyang University, Cheonan, Korea; 5 Department of Pediatrics, College of Medicine, Eulji University, Daejeon, Korea

## Abstract

Genetic diversity and antiviral activity for five common antiviral drugs of echovirus (ECV) 5 isolated in Korea have been described. The present study extended these tests to a Korean ECV 18 isolate. An outbreak of aseptic meningitis caused by the ECV 18 isolate was reported in Korea in 2005, marking the first time this virus had been identified in the country since enterovirus surveillance began in 1993. Using a sample isolated from stool specimen of a 5-year-old male patient with aseptic meningitis, the complete genome sequence was obtained and was compared it with the Metcalf prototype strain. Unlike the ECV5 isolate, the 3' untranslated region had the highest identity value (94.2%) at the nucleotide level, while, at the amino acid level, the P2 region displayed the highest identity value (96.9%). These two strains shared all cleavage sites, with the exception of the 2B/2C site, which was RQ/NN in the Metcalf strain but RQ/NS in the Korean ECV 18 isolate. In Vero cells infected with the Korean ECV 18 isolate, no cytotoxicity was observed in the presence of azidothymidine, acyclovir, amantadine, lamivudine, or ribavirin, when the drugs were administered at a CC_50 _value >100 μg/mL. Of the five drugs, only amantadine (IC_50_: 4.97 ± 0.77 μg/mL, TI: 20.12) and ribavirin (IC_50_: 7.63 ± 0.87 μg/mL, TI: 13.11) had any antiviral activity against the Korean ECV 18 isolate in the five antiviral drugs. These antiviral activity effects were similar with results of the Korean ECV5 isolate.

## Introduction

Human enteroviruses (HEVs), RNA viruses from the Picornaviridae family, comprise more than 80 immunologically-distinct serotypes that cause infections in humans. HEVs can be grouped as HEV-A to HEV-D and polioviruses. The HEV-B group containing echovirus (ECV) 18 consists coxsackieviruses B (CVB) 1 to 6, coxsackievirus A9, ECV 1 to ECV 7, ECV 9, ECV 11 to ECV 21, ECV 24 to ECV 27, ECV 29 to 33, enterovirus (EV) 69, and EV 73 [1-4].

ECVs cause the same types of infections in humans as the CVB group, but have been given a distinct classification primarily because they lack pathogenicity in newborn mice [5]. There are, however, strains of ECV that are pathogenic in mice [6]. The prototype strain of ECV 18, Metcalf, was isolated in 1995 from a patient with diarrhea in a sporadic case [7]. Thereafter, ECV18 was isolated from patients with exanthematous febrile disease and aseptic meningitis [8,9]. Generally, the clinical symptoms of HEVs, except for EV71, are asymptomatic or mild, usually with non-specific symptoms such as fever, irritation, agitation, sore throat, headache, myalgia, vomiting, mild abdominal discomfort, and diarrhea [10]. But, a fatality due to ECV 18 infection was described [11]. Recently, outbreaks of aseptic meningitis caused by ECV 18 have been frequently reported [12-15]. From 1970-2005, two peaks of ECV18 activity were observed in the United States (1986-1987 and 1995-2005), with the mortality rate being around 1.8% from 1983 to 2005 [16,17]. An outbreak of aseptic meningitis caused by ECV 18 occurred in Korea in 2005, marking the first time that ECV 18 had been identified in the country since enterovirus surveillance began in 1993 [18].

The HEV genome containing ECV 18 consists of an approximately 7,400 nucleotide (nt)-long single-stranded polar RNA molecule that is attached to a viral peptide (VPg) at the 5' end. The length of the 5' untranslated region (UTR) in the genome is about 700 nt, which is unusually long compared with the homologous region of cellular mRNA. The 5' UTR of HEVs harbors an internal ribosomal entry site (IRES) that fold to adopt a functional secondary RNA structure that drives translation initiation [19]. The coding region encompasses a single open reading frame (ORF) that encodes a polyprotein that can be divided into three sub-regions: P1, P2, and P3. P1 encodes the genetic information of four structural proteins: VP1-4. The non-structural proteins are encoded in P2 (2A-2C) and P3 (3A-3D). A short 3' UTR of approximately 100 nt separates the coding region from the poly (A) tail [6,20].

Currently, approximately 40 antiviral agents have been formally licensed for use in humans, mostly for treatment of infections caused by human immunodeficiency virus (HIV), hepatitis B virus (HBV), and herpes simplex virus (HSV). The list of antiviral agents licensed for use in treating highly-pathogenic RNA virus infections is very short, and includes anti-influenza medications, M2 channel inhibitors (amantadine and rimantadine), and neuramidase inhibitors (oseltamivir and zanaminir). Mechanism of azidothymidine and lamivudine as antiviral agent of HIV are termination of reverse transcription and viral DNA polymerase reaction, and acyclovir for HSV and varicella zoster virus acts as a termination of viral DNA polymerase reaction [21]. Ribavirin is licensed for the treatment of respiratory syncytial virus and hepatitis C virus infections [21,22]. Pleconaril was developed in 1996 for treatment of diseases associated with picornavirus infections and can be used in treatments against enterovirus and rhinovirus infections [23]. However, pleconaril use is extremely limited and development of resistance has been documented [24,25].

Previously, we explored the genetic diversity and antiviral activity for five common antiviral agents to ECV 5 isolated in Korea [26]. The present study extends these observations to a Korean ECV 18 isolate. Recently, outbreaks of aseptic meningitis by ECV 18 have been frequently reported, but information concerning the complete genome sequence is limited including in the GenBank database. In this study, the molecular biological characteristics and genetic diversity of Korean ECV 18, which is widespread and for which no antiviral agents are known, were analyzed through complete nucleotide sequencing and comparison with the Metcalf prototype ECV 18 strain. Five compounds capable of inhibiting viral reproduction (azidothymidine, acyclovir, amantadine, lamivudine, and ribavirin) were tested for antiviral activity for Korean ECV 18.

## Materials and methods

### Virus isolation and identification

The Korean ECV 18 strain was isolated from a stool sample of a male patient with aseptic meningitis who had been hospitalized to the Department of Pediatrics at Soonchunhyang University Cheonan Hospital in April 2005. The pretreated specimen was inoculated into Vero cells and incubated at 37°C in an atmosphere of 5% CO_2 _until the appearance of cytopathic effects (CPE). The identification of the Korean isolate was verified by a Basic Local Alignment Search Tool search of VP1 sequences at the National Center for Biotechnology Information website http://www.ncbi.nlm.nih.gov/. The VP1 sequences of Korean isolate had the highest nucleotide similarity with ECV 18 serotype strains [3].

### Nucleotide sequencing and sequence analysis

The complete nucleotide sequence of the Korean ECV 18 strain was determined using a primer walking strategy; the sequences of the genome termini were determined by random amplification of cDNA ends (RACE) system (Invitrogen, Carlsbad, CA, USA). Polymerase chain reaction (PCR) products were purified using a QIAquick PCR Purification Kit (Qiagen, Valencia, CA, USA). The purified DNA was added to a reaction mixture containing 2 μL of terminator reaction mix (ABI Prism BigDye Terminator Cycle Sequencing Kit; Applied Biosystems, Foster, CA, USA) and 2 pmole of each primer. Sequencing involved an initial denaturation at 96°C for 1 min and 25 cycles consisting of 96°C for 10 sec, 50°C for 5 sec, and 60°C for 4 min in a Gene Amp PCR system 2700 (Applied Biosystems). The products were purified by precipitation with 100% cold ethanol and 3 M sodium-acetate (pH 5.8), then loaded on an automated 3100 Genetic Analyzer (Applied Biosystems). Nucleotide sequences of ECV 18 Korean isolates were constructed to contig and were compared with the reference Metcalf strain (Accession no. AF317694). The Metcalf nucleotide sequence was obtained from the GenBank database [27]. Sequence analyses were performed using computer software included in the Lasergene package (DNASTAR, Madison, WI, USA).

### Antiviral drugs and antiviral activity assays

Assays of antiviral activity and cytotoxicity involved the modified sulforhodamine B (SRB) assay [28-30]. Ribavirin was purchased from DUCHEFA (Haarlen, Netherlands). Azidothymidine, acyclovir, amantadine, lamivudine, and sulforhodamine B (SRB) were purchased from Sigma-Aldrich (St. Louis, MO, USA).

One day before infection, Vero cells were seeded (2 × 10^4 ^cells/well) in wells of a 96-well plate. The next day, the medium was removed from each well and the adherent cells were washed with 1 × phosphate buffered saline (PBS). Infectivity of the virus stock was determined by the SRB assay [28-30] and was determined as infectivity of the ECV 18 by SRB ID_50 _(50% infective dose). Subsequently, 0.09 mL of diluted virus suspension of ECV 18 containing CCID_50 _(50% cell culture infective dose) of the virus stock to produce an appropriate CPE within 2 days after infection and 0.01 mL of medium containing an appropriate concentration of the compounds were added. The antiviral activity of each agent was determined with a 10-fold diluted concentration ranging from 0.1-100 μg/mL. Three wells were used as ECV 18 controls (virus-infected non-drug-treated cells), while three wells were used as cell controls (non-infected, non-drug-treated cells). The culture plates were incubated at 37°C in 5% CO_2 _for 2 days. After washing once with 1 × PBS, 100 μL of cold (-20^o^C), 70% acetone was added to each well and left for 30 min at -20^o^C. The acetone (70%) was discarded and each 96-well plate was dried in an oven for 30 min. 100 μL of 0.4% (w/v) SRB in 1% acetic acid was added to each well and left at room temperature for 30 min. Unbound SRB was removed and the plates were washed five times with 1% acetic acid before oven-drying prior to being left in the drying oven for one day. Bound SRB was solubilized with 100 μL of 10 mM unbuffered Tris-base solution and each plate was left on a table for 30 min. The absorbance was read at 540 nm using a VERSAmax microplate reader (Molecular Devices, Palo Alto, CA, USA) with a reference absorbance at 620 nm. To calculate the IC_50 _(50% inhibitory concentration) values, the results were transformed to percentage of controls and the IC_50 _values were graphically obtained from the dose-response curves. The percent protection achieved by the test compound in ECV 18-infected cells was calculated by the following formula:

$$\frac{{\left(\text{O}{\text{D}}_{\text{t}}\right)}_{\text{ECV}18}-{\left(\text{O}{\text{D}}_{\text{c}}\right)}_{\text{ECV}18}}{{\left(\text{O}{\text{D}}_{\text{c}}\right)}_{\text{moc}{\text{k}}^{-}}{\left(\text{O}{\text{D}}_{\text{c}}\right)}_{\text{ECV}18}}\times 100$$

where (OD_t_)_ECV 18 _is the optical density measured with a given concentration of the test compound in ECV 18-infected cells, (OD_c_)_ECV 18 _is the optical density measured for the control untreated ECV 18-infected cells, and (OD_c_)_mock _is the optical density measured for control untreated mock-infected cells. The therapeutic index was defined as 50% cytotoxic concentration (CC_50_)/IC_50_.

### Cytotoxicity assay

Vero cells were seeded into wells of a 96-well culture plate at a concentration of 2 × 10^4 ^cells/well. The next day, the medium was discarded and the medium in each well was replaced with medium containing the serially-diluted drugs, and the cells were incubated for another 48 hours. The culture medium was removed and the adherent cells were washed with 1 × PBS. The next step assessed antiviral activity as described above. To calculate the CC_50 _values, the results were transformed to percentage of controls and the CC_50 _values were graphically obtained from the dose-response curves.

## Results

### Complete nucleotide sequence of Korean ECV18

The genome of the Korean ECV 18 isolate was sequenced (GenBank accession no. HM777023) and its amino acid sequence was deduced. The genome was 7,413 nt in length, excluding the poly(A) tail. The 5' UTR contained 743 nt, followed by an ORF that encoded a viral polyprotein consisting of 2,189 codons, between a start codon (AUG) at position 744 and a stop codon (UGA) at position 7,310. The 3' UTR was 103 nt in length.

### Genome comparison between the Korean ECV18 isolate and the Metcalf strain

The Korean ECV 18 isolate genome was divided into five regions (5' UTR, P1, P2, P3, and 3' UTR) and aligned with the Metcalf strain using Megalign. The 3' UTR region had the highest level of nucleotide identity (85.3%), followed by the P3 region (82.2%), 5' UTR (82.0%), P2 (81.2%), and the P1 region (79.7%). The P2 region displayed the highest level of amino acid identity (96.9%), followed by the P3 region (96.8%), and the P1 region (95.2%) (Table 1). Most of the cleavage sites were identical between the Korean ECV 18 isolate and the Metcalf strain. The only exception was the cleavage site between 2B and 2C, which was RQ/NN in the Metcalf strain and RQ/NS in the Korean ECV 18 isolate (Table 2).

**Table 1:** Comparison of identities in nucleotide and amino acid sequence between the Korea ECV 5 and 18 isolates with each prototype strain

		5'UTR	P1	P2	P3	3'UTR
ECV 5*	Nucleotide	81.8	85.3	80.0	84.8	84.5
	Amino Acid	-	97.7	96.9	98.0	-

ECV 18	Nucleotide	82.0	79.7	81.2	82.2	94.2
	Amino Acid	-	95.2	96.9	96.8	-

* The results of ECV 5 were reported by Park *et al. *[26].

ECV5 and ECV18 are compared with Noyce and Metcalf strains, respectively.

**Table 2:** Comparison of predicted N-terminal cleavage sites for the Korean ECV 5 and 18 isolate with each prototype strain

Region		ECV 5*		ECV 18	
			
		Noyce	Kor-ECV5	Metcalf	Kor-ECV18
P1	VP4	-	-	-	-
	VP2	LN/SP	LN/SP	LN/SP	LN/SP
	VP3	PQ/GL	PQ/GL	TQ/GV	TQ/GV
	VP1	LQ/GD	LQ/GD	LQ/GD	LQ/GD
P2	2A	TY/GA	TR/GA	TH/GA	TH/GA
	2B	EQ/GV	EQ/GV	EQ/GV	EQ/GV
	2C	RQ/NN	RQ/NN	RQ/NN	RQ/NS
P3	3A	FQ/GP	FQ/GP	FQ/GP	FQ/GP
	3B	FQ/GA	FQ/GA	FQ/GA	FQ/GA
	3C	VQ/GP	VQ/GP	VQ/GP	VQ/GP
	3D	EQ/GE	EQ/GE	EQ/GE	EQ/GE

* The results of ECV 5 were reported by Park *et al. *[26].

ECV5 and ECV18 are compared with Noyce and Metcalf strains, respectively.

### Antiviral activity of Korea ECV18

**Figure 1:**
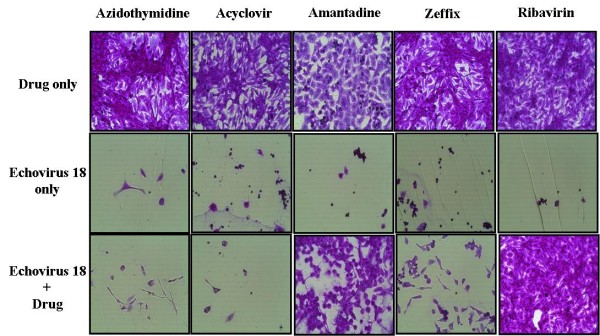
**Efficacy of antiviral agents at preventing cytopathic effects of Vero cells induced by infection with Korean ECV 18**. The concentration of each drug is commonly 100 μg/mL, and time of reaction is 72 hours.

No signs of cytotoxicity were observed in Vero cells treated with any of the five antiviral agents at a CC_50 _value >100 μg/mL. Amantadine and ribavirin exhibited antiviral activity against the Korean ECV 18 strain, while azidothymidine, acyclovir, and lamivudine did not. Amantadine displayed an IC_50 _value of 4.97 ± 0.77 μg/mL and a TI value of 20.12, while ribavirin displayed an IC_50 _value of 7.63 ± 0.87 μg/mL and a TI value of 13.11 (Table 3 and Figure 1).

**Table 3:** Efficacy of antiviral drugs against Korean ECV 18

Compounds	IC_50_^a^	CC_50_^b^	TI^c^
Azidothymidine	ND^d^	>100	-
Acyclovir	ND^d^	>100	-
Amantadine	4.97 ± 0.77	>100	20.12
Lamivudine	ND^d^	>100	-
Ribavirin	7.63 ± 0.87	>100	13.11

These results represent mean ± S.D (n = 3).

^a ^Concentration of compound in μg/mL producing 50% inhibition of virus-induced cytopathic effects.

^b ^The 50% cytotoxic concentration for target cells in μg/mL.

^C ^Therapeutic index (TI) = CC_50_/IC_50_

^d ^Not Determined

## Discussion

This is the first report that describes the complete nucleotide sequence for an ECV 18 isolated in Korea. A previous study demonstrated a relatively high sequence identity between different ECV serotypes for the 5'UTR sequence, moderate for the P2 and P3 regions, and lowest for the P1 region [31]. However, this pattern was different from the comparison between the Korean ECV 18 isolate and the Metcalf strain, in which the 3'UTR had the highest nucleotide sequence identity (94.2%), while the identities of the other regions were relatively low (>82.2%). Amino acid sequences for the functional protein coding regions had much higher sequence identities (>96.8%).

Generally, it has reported that about a 20% genetic difference between the Metcalf prototype and the current widespread strain exists, mainly at the cleavage site. Therefore, development and screening of antiviral drugs have to be focused on the object of the current epidemic strain [28]. Cleavage site variations have often been reported for VP2/VP3, VP3/VP1, and VP1/2A [31,32]. However, presently a substitution (RQ/NN→RQ/NS) at 2B/2C in ECV 18 was observed. Cleavage site variations between ECV 5 and ECV 18 were evident in P1 of the capsid protein coding region, rather than P2 and P3 of non-structural protein coding regions.

Of the five antiviral drugs presently-tested (azidothymidine, acyclovir, amantadine, lamivudine, and ribavirin), only amantadine (IC_50_: 4.97 μg/mL) and ribavirin (IC_50_: 7.63 μg/mL) displayed antiviral activity against Korean ECV 18, with amantadine showing stronger effects than ribavirin. The same results were obtained with the ECV 5 antiviral activity test. Therefore, amantadine and ribavirin could be applied to patients infected with ECV 18 as well as ECV 5 infection. Amantadine suppresses the IRES mediated translation and ribavirin is a nucleoside analogue with broad-spectrum antiviral activity by decreasing viral replication in EV71 [33,34].

In conclusion, this manuscript is the first report of the complete nucleotide sequence of the Korean ECV 18 strain, as well as the first examination of its response to various antiviral agents. This data should be useful in preventing future outbreaks of ECV 18 and in treating patients infected with the strain. Accordingly, it is necessary to screen the activities of the same kind of antiviral agents to the various enterovirus serotypes and reveal the antiviral mechanisms.

## Competing interests

The authors declare that they have no competing interests.

## Authors' contributions

KSP, SGY and KAB performed complete sequencing and antiviral tests. DSC and YJC contributed to collection specimen and clinical diagnosis. JSP and SJL designed the study and critically revised the manuscript. All of the authors read and approved the final version of the manuscript.
